# Application of lipid-based nanoparticles in cancer immunotherapy

**DOI:** 10.3389/fimmu.2022.967505

**Published:** 2022-08-08

**Authors:** Zhongkun Zhang, Siyu Yao, Yingwen Hu, Xiaobin Zhao, Robert J. Lee

**Affiliations:** ^1^ Division of Pharmaceutics and Pharmacology, College of Pharmacy, The Ohio State University, Columbus, OH, United States; ^2^ Department of Food Science and Technology, The Ohio State University, Columbus, OH, United States; ^3^ The Whiteoak Group, Inc., Rockville, MD, United States

**Keywords:** lipid-based nanoparticle, drug delivery, cancer immunotherapy, cell and gene therapy, nanomedicine

## Abstract

Immunotherapy is revolutionizing the clinical management of patients with different cancer types by sensitizing autologous or allogenic immune cells to the tumor microenvironment which eventually leads to tumor cell lysis without rapidly killing normal cells. Although immunotherapy has been widely demonstrated to be superior to chemotherapies, only a few populations of patients with specific cancer types respond to such treatment due to the failure of systemic immune activation. In addition, severe immune-related adverse events are rapidly observed when patients with very few responses are given higher doses of such therapies. Recent advances of lipid-based nanoparticles (NPs) development have made it possible to deliver not only small molecules but also mRNAs to achieve systemic anticancer immunity through cytotoxic immune cell activation, checkpoint blockade, and chimeric antigen receptor cell therapies, etc. This review summarized recent development and applications of LNPs in anticancer immunotherapy. The diversity of lipid-based NPs would encapsulate payloads with different structures and molecular weights to achieve optimal antitumor immunity through multiple mechanisms of action. The discussion about the components of lipid-based NPs and their immunologic payloads in this review hopefully shed more light on the future direction of anticancer immunotherapy.

## Introduction

**Figure 1:**
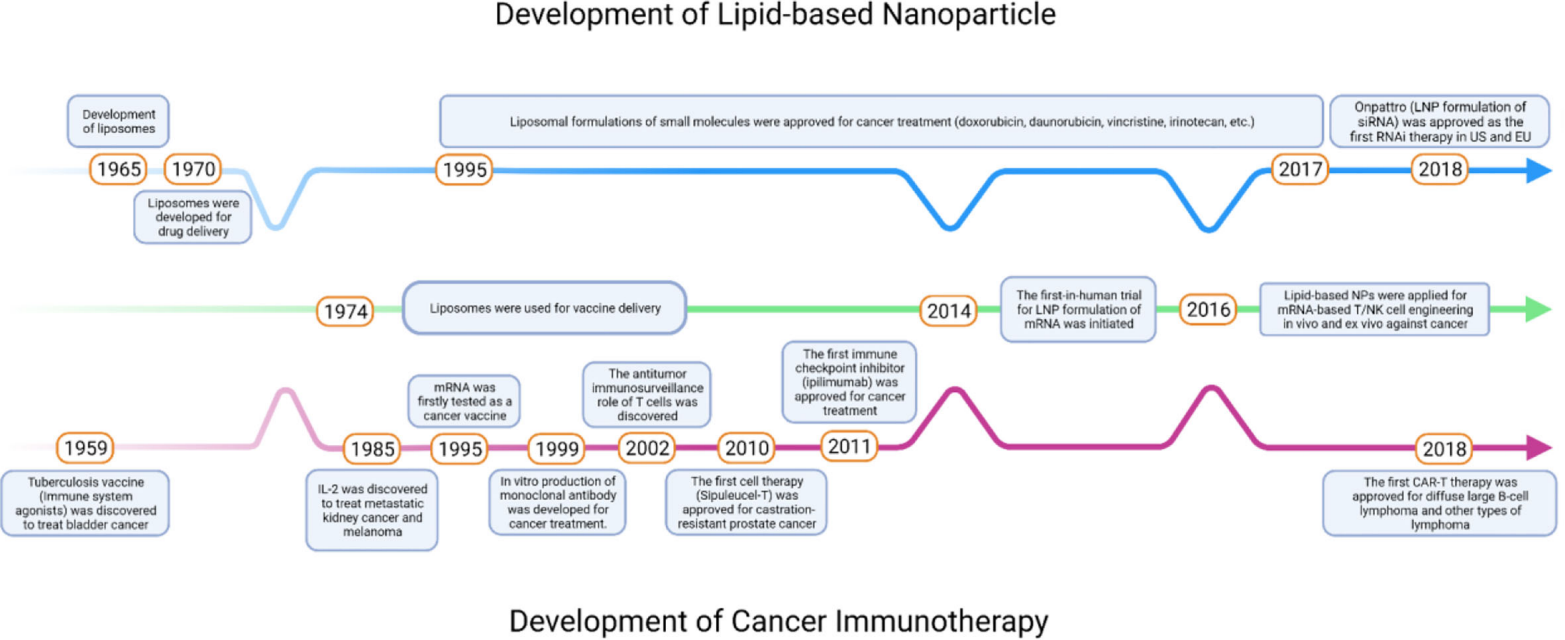
Timeline of key milestones throughout the development of lipid-based NPs and cancer immunotherapies.

Over the past decade, many immunotherapeutic agents have been developed against various types of cancer in clinic trials (**Figure 1**). The history of cancer immunotherapy started with using attenuated bacteria, tuberculosis vaccine Bacille Calmette-Guérin (BCG), to prevent the recurrence of invasive bladder cancer (1). Later, Dunn et al. suggested that lymphocytes played a role of immunosurveillance in tumor microenvironments (TMEs) (2). However, at the time the lack of methods to detect tumor-specific antigens and the difficulties to culture lymphocytes *in vitro* limited its application in the cancer immunotherapy. The first anticancer immunotherapeutic agent was used T cell growth factor interleukin 2 (IL-2), where IL-2 treatment showed significant tumor suppression by enhancing T-cell activation in patients with metastatic kidney cancer and melanoma (3). In the late 1990s, the production of monoclonal antibodies (mAbs) using hybridomas has been well established which led to the development of antibody-based therapeutics targeting tumor specific surface markers (4). Recently, there have been thriving efforts in the immunostimulatory small molecules, immune checkpoint inhibitors (ICIs) targeting immune cells, autologous T cells or natural killer (NK) cells expressing chimeric antigen receptors (CARs), and mRNAs expressing tumor antigens or CARs for cancer immunotherapy (5–7). Among these, small molecules, ICIs, and mRNA therapies are used as stand-alone treatments for many solid tumors such as melanoma, non-small cell lung cancer (NSCLC), and urothelial carcinoma (5, 8), whereas CAR T cell therapies exhibit distinct clinical responses in patients with blood cancer (9).

**Figure 2:**
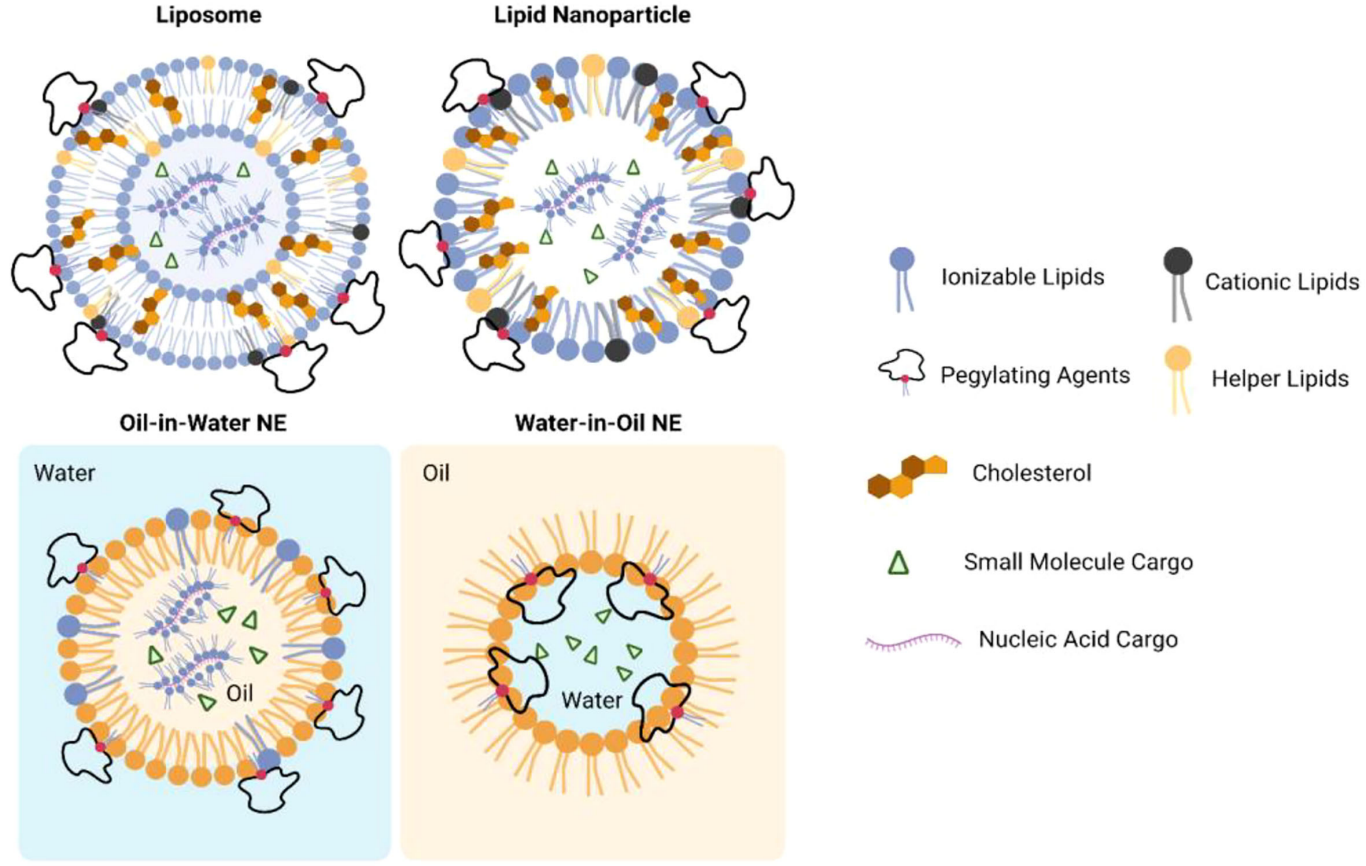
Structures of lipid-based nanoparticles when encapsulating small molecule or nuclei acid cargos.

Despite the great promise of immunotherapy against cancer, there also have been significant limitations with these immunotherapies: Poor water solubility, high immune-mediated adverse events (irAEs), and loss of bioactivity after long-term administration have limited the immunostimulatory efficacy of small molecules therapeutics (10). Unfortunately, these drawbacks are also interactional. Poor water solubility and off-target effects may lose the bioactivity of drugs and reduce the responses in patients’ bodies, and patients may develop irAEs when physicians decide to increase the dose. Therefore, the main challenges for cancer immunotherapies can be attributed to the lack of delivery systems which can keep the therapeutic payloads accessible to their targets. Lipid-based nanoparticles (NPs), including liposomes, lipid nanoparticles (LNPs), and nanoemulsions (NEs), have been developed as promising platforms to deliver a variety of therapeutic agents (11, 12) (**Figures 1**, **2**). Compared to other nanosized delivery systems, lipid-based NPs are superior in minimizing systemic toxicity while maintaining high solubility in the water phase that neither polymeric NPs nor inorganic NPs could overcome for clinical use (13), and these advantages make lipid-based NPs the most common type of FDA-approved nanomedicines (14). Different structural designs endow lipid-based NPs with enhanced physical stabilities and the capacities of loading cargos with different sizes and hydrophobicity. From the 1960s to the 1970s, liposomes were first developed to deliver active pharmaceutical ingredients with simple vesicular formulations composed of an aqueous interior core with lipid bilayers (15–17). Later, different designs of LNPs were introduced with similar liposomal structures but resemble more micelle-like structures. The more complex internal lipid architectures in LNPs make them more suitable for encapsulating genetic payloads with long-term stability (18). NEs are biphasic systems in which oil and water phases are mixed in the form of nano-sized droplets (19). While water-in-oil NEs are important ingredients for topical applications, oil-in-water NEs were developed and preferred as an alternative system to liposomes or LNPs with micelle-like structures but covered by oil droplets (20). NEs are thermodynamically unstable compared with liposomes and LNPs which result in phase separation during long-term storage. However, NEs can be optimized to be kinetically stable with the help of surfactants and helper lipids to make the NEs more potent for encapsulating hydrophobic drugs (21). This review will discuss in detail the components of lipid-based NPs and provide an overview of the current applications of LNPs in delivering anticancer immunotherapies which may shed light on the future direction of anticancer immunotherapy.

## Components of lipid-based NPs

Lipid-based NPs exhibit various types of structure. Majority of the lipid-based NPs exhibited near spherical shape with one or more lipid external layers (**Figure 2**). Although liposomes, LNPs, and NEs may exhibit different internal architectures, typical lipid-based NPs are composed of cationic lipids or ionizable lipids with tertiary or quaternary amines to encapsulate anionic payloads; helper lipids also were used to stabilize the lipid layer and facilitate membrane fusion; polyethylene glycol (PEG) lipids or surfactants were added to improve colloidal stability for long-term storage and prevent rapid degradation of payloads when administered into systemic fluids (18, 22). In addition, NEs would also include an oil phase which may be tri-, di-, or mono-acylglycerols, vegetable oils, mineral oils, free fatty acids, etc. (23). Representative structures of each component in lipid-based NPs are shown in **Figure S1**.

### Cationic and ionizable lipids

Drug encapsulation in lipid-based NPs was initially relied on the passive methods using zwitterionic lipids. It showed relatively poor encapsulation efficiencies (<40%) and low transfection potency for gene delivery (24, 25). The development of N-[1-(2,3-dioleyloxy) propyl]-N,N,N-trimethylammonium chloride (DOTMA) significantly enhanced the anionic drug encapsulation through electrostatic interactions (26). However, this first generation of such “lipoplexes” is highly unstable with broad size distributions and therefore have not been proven effective for *in vivo* delivery purposes. 1,2-dioleoyl-3-trimethylammonium-propane (DOTAP), a biodegradable analogue of DOTMA, was then developed for lipid-based NPs gene delivery (27). Dimethyldioctadecylammonium bromide (DDA), a quaternary ammonium lipid, was later used in a gene delivery system as well as an immune adjuvant due to its special immunogenicity (28). The first trial of lipid-based NPs containing a cationic lipid was to encapsulate plasmid DNA (pDNA) using dioctadecyl-dimethyl-ammonium chloride (DODAC) (29). The author demonstrated that the injection of lipid/pDNA particles showed the halflife (t_1/2_) of pDNA was extended to 7 h in mice compared to naked pDNA, which had a t_1/2_ of less than 10 min. The prolonged circulation time of lipid/pDNA particles in mice resulted in dose accumulation in distal tumors over 24 h with minor accumulation in liver and spleen (30). With the development of lipid-based NPs, DOTMA and DOTAP have been successfully applied in mRNA delivery as cancer vaccines (31–34).

Ionizable lipids were a series of lipids with native pKa values below 7. It was designed to enhance anionic drug encapsulation and minimize systemic toxicities by cationic lipids due to permanent positive charges. Lipid-based NPs embedded with ionizable lipids could load anionic payloads at pH values below the pKa of ionizable lipids when it is positively charged. Then the drug-loaded lipid-based NPs are suitable for systemic delivery with a neutral exterior depending on physiological pH values (35). When trapped in endosomes after cellular uptake, where pH is lower than physiological pH, ionizable lipids are protonated and facilitate membrane destabilization which eventually releases payloads from lipid-based NPs into the intracellular compartment (35, 36). Researches showed that the optimal pKa value for ionizable lipids is around 6.5 for nucleic acid delivery in the murine FVII model (37). Aside from the pKa value, the linker between the ionizable group and alkyl chains is another factor for optimized gene delivery, where ketal linker was demonstrated to be more potent than ester and alkoxy linkers (38). Therefore, the potency of gene delivery relies considerably on the optimization of ionizable lipids in lipid-based NPs.

1,2-dioleoyl-3-dimethylammonium propane (DODAP) and 1,2-dioleyloxy-N,N-dimethyl-3-aminopropane (DODMA) were firstly developed for antisense oligonucleotides (ASOs) delivery with significantly increased circulation half-lives of ASOs *in vivo* through i.v. administration (39). Later, Dilinoleyl-DMA (DLinDMA), a dilinoleoyl analogue of DODMA, was introduced for silencing apolipoprotein B (ApoB) in the liver following i.v. administration (40). In another study using a murine factor VII (FVII) model for hepatic gene delivery, 2,2-dilinoleyl-4-(2-dimethylaminoethyl)- (1,3)-dioxolane (DLin-KC2-DMA, or KC2) demonstrated higher potency in delivering FVII siRNA compared with DLinDMA (38). Recent lipid screening study identified a new candidate heptatriaconta-6,9,28,31-tetraen-19-yl-4-(dimethylamino)butanoate (DLin-MC3-DMA or MC3) (37). MC3 exhibited superior siRNA delivery targeting TTR mRNA with ED_50_ of 0.005mg/kg (37). Under the hot trend of gene therapy, more ionizable lipids have been introduced for gene delivery specifically. Recent studies revealed that new classes of ionizable lipids have better gene delivery efficacy and pharmacokinetics than MC3, as summarized in **Table S1** (41–54).

### Helper lipids and other components

Helper lipids determine the shape of lipid-based NPs based on their different polymorphic phase tendencies, which also play an important role in intracellular delivery (55, 56). For example, lipids with the large hydrophilic heads are “cone-shaped” which tend to form micelle-like structures. Lipids with cylindrical geometry, such as phosphatidylcholine (PC), are compatible with bilayer structures. Lipids containing unsaturated acyl chains, such as unsaturated phosphatidylethanolamine (PE), have fixed geometry and are likely to form the inverted hexagonal (H_II_) phase (57).

Lipid bilayers formed by helper lipids can be stabilized by cholesterol where cholesterol can fill the geometry gaps between phospholipids (58). The inclusion of cholesterol in lipid-based NPs results in great stability in the presence of serum proteins. In addition, cholesterol is also able to facilitate membrane fusion which favors delivering nucleic acid cargos (59), while high percentages of cholesterol in lipid-based NP formulation may end up destabilizing lipid layers (60). Therefore, the trade-off between colloidal stability and transfection activity needs to be optimized when combining cholesterol and phospholipids in lipid-based NPs formulation.

Fusogenic oils are special components in NEs which is made of fatty acids, lipid substitutes, waxes, oil-soluble vitamins, and other lipophilic components. The viscosity, refractive index, density, phase behavior, and interfacial tension of the oils greatly determine the stability and functional properties of NEs (61). Other function of oils in NE-based drug delivery is to solubilize hydrophobic agents such as poor water-soluble chemotherapies and immunomodulatory agents (62, 63). It has been reported that some of the fusogenic oils (such as medium-chain oils, long-chain oils, and squalene) may stimulate innate immune system by activating toll-like receptors (TLRs) (64–66). There is no direct evidence showing that oil-induced innate immune activation could lead to antitumor responses *in vitro* or *in vivo*. Nonetheless, preclinical immunogenicity studies are recommended when developing nanomedicines containing fusogenic oils.

PEG lipids or surfactants are the most commonly applied non-ionic hydrophilic stabilizer for lipid-based NPs (67, 68). PEG lipids reduce the tendency of particle aggregation by steric stabilization, thereby enhancing formulation stability and prolonging the blood circulation time by reducing clearance mediated by the kidneys and mononuclear phagocyte system (MPS) (67, 68). 1,2-dimyristoyl-rac-glycero-3-methoxypolyethylene glycol-2000 (PEG2000-DMG) and 1,2-distearoyl-rac-glycero-3-methoxypolyethylene glycol-2000 (PEG2000-DSG) have been widely applied in lipid-based NPs for gene delivery (68). It also showed that LNPs containing PEG2000-DMG exhibit higher delivery efficiency *in vivo* than LNPs containing PEG2000-DSG, though PEG2000-DSG could prolong circulation times for LNPs in blood compared to PEG2000-DMG (68). Such differences may be attributed to the dissociation of PEG2000-DMG within LNPs compared with PEG2000-DSG, which could better facilitate cellular uptake and endosomal escape (68). In NEs, PEG lipids work as an emulsifier to improve the particle stability when the oil and water phase are mixed to yield a temporary emulsion (19). It should be noted that higher amount of PEG lipids in the formulation may encounter potential side effects such as PEG-specific immunogenicity, changes in pharmacokinetic behavior, potential toxicities of PEG lipids or side-products, etc. (67). In addition, too much of PEG lipids also may jeopardize the intracellular cargo release for gene delivery (69). Therefore, scientists in the field usually use less than 10% (molar ratio) of PEG lipids in their formulation (70).

## Small molecule-based immune activation

### Chemoimmunotherapies

Lipid-based NPs were firstly applied to encapsulate chemotherapies as anticancer therapeutics. Conventional anticancer chemotherapies were approved for cancer treatment based on their direct cytotoxic damage to cell proliferation, accumulating evidence indicates that the host immune system also plays an important role during the therapeutic process of chemotherapies which eventually leads to antitumor responses (71).Here are some examples of using Lipid-based NPs for the delivery of chemotherapeutics. Among all the chemotherapies approved or under clinical investigation, doxorubicin has been demonstrated to preferentially deplete myeloid-derived suppressor cells (MDSCs) in a murine breast cancer model (72). In addition, several studies revealed that doxorubicin could trigger dendritic cells (DCs) to release IL-1β which further activate IL-17 producing γδ T cells and recruit anti-tumoral IFN-γ expressing CD8+ T cells into TME (73–75). Similar to doxorubicin, daunorubicin and mitoxantrone treatments also stimulate the innate immune system and eventually lead to a major influx of myeloid and lymphoid cells into TME (76–78). Vincristine can upregulate PD-L1 and sensitize tumor antigen presenting in TME which can be a sensitizer before PD-L1 blockade therapy or DC-based immunotherapy (79, 80). Similar ability was also observed in irinotecan which could deplete regulatory T cells (Tregs), upregulate major histocompatibility complex 1 (MHC1) and PD-L1, turning TME more sensitive to immunotherapy (81). Treatment with topotecan, another camptothecin derivative to irinotecan, also has upregulated MHCI and Fas, making topotecan-treated tumors additive to effector T cell killing (82). *In vitro* exposure of cisplatin to melanoma cell lines resulted in the expression of T-cell chemokines such as CCL5, CXCL9, and CXCL10 (83). In clinical trials, a significant increase of tumor-infiltrating lymphocytes (TILs) was observed in 7 out of 21 breast cancer patients who received the first dose of paclitaxel (84). Other *in vivo* studies reported the increase of tumor MHCI and paclitaxel-dependent CD8+/CD4+ T cells infiltrated in a murine ovarian cancer model (85). Paclitaxel was also demonstrated to preferentially deplete Tregs (86), promote MDSCs into a more DC-like phenotype (87), activate TLR4, and shift macrophage polarization to a pro-inflammatory phenotype (88). Lastly, treatment of oxaliplatin in a murine colorectal cancer model resulted in upregulated tumor MHCI, and decreased immune suppressor cells (Tregs, MDSCs, and tumor-associated macrophages, TAMs) (89). Interestingly, the liposomal formulation of oxaliplatin exhibited superior antitumor immunity compared to free oxaliplatin, suggesting that accurate delivery of chemotherapies to TME would trigger better antitumor immunity (89).

### Immune system agonists (small molecule, nucleic acid, or peptide only)

**Figure 3:**
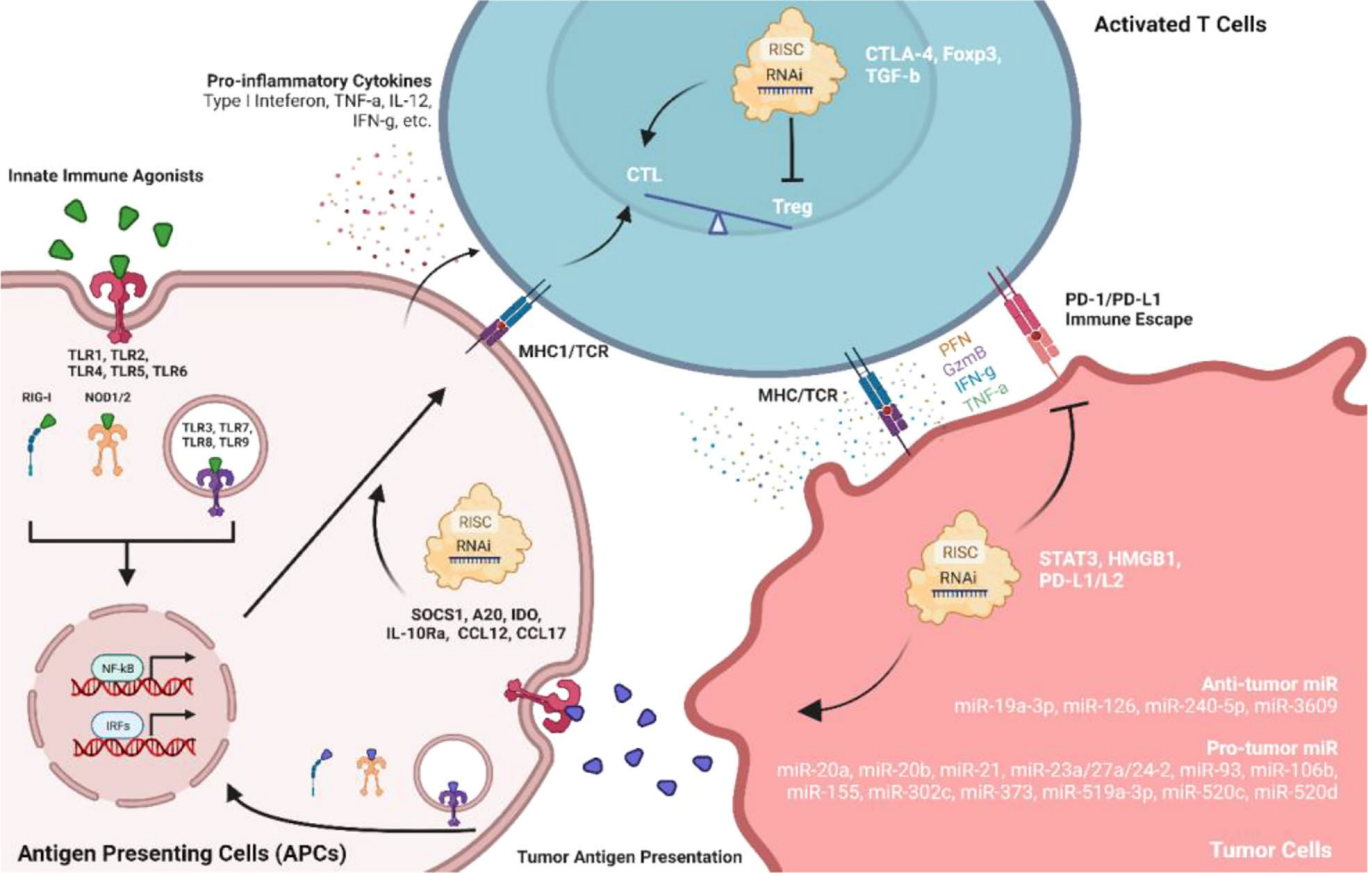
Role of innate immune agonists and RNAi in facilitating antitumor immune responses among APCs, T cells, and TMEs. Agonists of pattern recognition receptors (PRRs) on antigen-presenting cells can directly activate the immune system against tumors. Meanwhile, RNAi therapeutics targeting immunomodulatory factors or non-coding RNAs may also facilitate antitumor immunity.

Many evidence from chemotherapy-based immunomodulation revealed that successful antitumor immunity requires cross-function between innate and adaptive immune activations. Antigen-presenting cells (APCs, either dendritic cells (DCs) or macrophages) serve to early detect cancers by sensing tumor-associated antigens or pattern-/damage-associated molecular patterns (PAMPs/DAMPs) through pattern recognition receptors (PRRs) (90). Activated APCs will trigger pro-inflammatory cytokines and chemokines to employ the adaptive immune system where antigen-specific T cells will be activated to direct tumor cell killing (**Figure 3**) **(** 91). Antitumor adjuvants have been developed as tumor antigen simulators, ligands for PRRs or other PAMP/DAMP-associated pathways. Agonists for TLRs, NOD-like receptors (NLRs), and RIG-I-like receptors (RLRs) are the adjuvants used to induce pro-inflammatory immune responses which favor anti-tumor activities. Among TLR agonists, Pam3Csk4, a TLR1/2 agonist, was successfully developed using LNPs with OVA mRNA against a murine lymphoma model (92). Poly (I:C), a TLR3 agonist, was capable to be incorporated in not only lipid-based NPs (93, 94) but also inorganic NPs (95). TLR7/8/9 agonists are widely studied as antitumor adjuvants. Imidaziquinoline-like TLR 7/8 agonists, including imiquimod and resiquimod (R848), was loaded into the liposomal formulation to prolong its retention time in blood circulation (96). When considering the poor water solubility of TLR7/8 agonists, NEs formulation is also a suitable delivery platform to improve drug solubility thus the drug loading (63). Synthetic TLR9 agonists are oligonucleotides (ODNs) with cytosine-phosphate-guanine (CpG) motifs. CpG ODNs can be easily incorporated into tumor-targeting lipid-based NPs with cationic or ionizable lipids through electrostatic interactions (97–100). Agonists for RLRs are also nucleic acid-based dsRNAs (101). Studies have shown that dsRNA targeting RLRs could be encapsulated into lipid-based NPs and polymeric NPs for systemic delivery in murine pancreatic cancer and breast cancer models (102, 103). In a study comparing the antitumor immunity carried out by liposomal TLR agonists and NLR agonists, NLR agonists also exhibit early antitumor activity (104). However, the antitumor activity of NOD1 agonist was not associated with DC-driven adaptive immune responses, suggesting that NOD1 activation would trigger an alternative cytotoxic adaptive immune response against tumor which differs from TLR agonists. Another liposomal-based innate immune agonist, mifamurtide, has been demonstrated to target both TLR4 and NOD2 which synergize NF-kB activation and pro-inflammatory tumoricidal activities (105). Stimulator of interferon genes (STING) is another type of PRR which located in the endoplasmic reticulum. Activated STING would potentiate type I interferons with other pro-inflammatory cytokines which may enhance antitumor immunity (106). Cyclic dinucleotides (CDNs), including cyclic guano-sine monophosphate–adenosine monophosphate (cGAMP), are common agonists for STING receptors which can be encapsulated into lipid-based NPs to potentiate systemic antitumor immunity (107–110). Since STING agonists require intracellular delivery to the endoplasmic reticulum, lipid-based NPs delivery system requires an optimized fusogenic activity for efficient endosomal escape.

### RNA interference (RNAi) technologies

RNAi technologies (siRNA, shRNA, miRNA, ASO, etc.) can induce specific gene regulation which became a new therapeutic area in infectious diseases, neurodegenerative disorders, cancer, and other rare diseases. Besides direct targeting of specific oncogenes, RNAi could also potentiate antitumor immune responses by downregulating immune-suppressive proteins (**Figure 3**). Studies reported that elevated activity of signal transducer and activator of transcription 3 (STAT3) is positively associated with tumor progression. Knockdown of STAT3 using siRNA successfully inhibits tumor growth with increased tumor infiltration of CD8+ T cell and NKT cell in murine melanoma models (111, 112). STAT3 downregulation also significantly increased pro-inflammatory cytokine production such as IFN-g, IL-12, and TNF-a (112). Suppressor of cytokine signaling 1 (SOCS1) negatively regulates antigen presentation process. Downregulation of SOCS1 by siRNA enhanced antigen-specific DC maturation with elevated expression of IFN-g, IL-12, and decreased IL-4 which potentiate Th1 cell differentiation and antitumor immunity (113). Similar activities were also observed using siRNA to downregulate A20 and indoleamine 2, 3-dioxygenase (IDO) in DCs (114–117). A20 siRNA could inhibit CD4+ CD25+ Tregs suppression which further enhanced CD8+ T cells immune responses in TMEs (118). Programmed dead ligand 1 (PD-L1) is another exciting target that facilitates tumor immune escape from host immune systems. siRNA against PD-L1 can preferentially transfer CD11c+ PD-L1+ tolerogenic DCs into potent stimulators of CTLs in TMEs through PD-L1 downregulation and multiple TLRs activation (119). The antitumor efficacy of siRNA against PD-L1 can be enhanced by co-inhibiting with CTLA-4 (120). Another study also indicated that siRNA against PD-L1 or PD-L1 improved effector functions of tumor-specific CD4+ and CD8+ T cells *in vitro* (121), suggesting that PD-L1/PD-L2 siRNA is an exciting strategy to inhibit the immune suppressive tumor-specific T cells. High-mobility group box 1 (HMGB1) is highly expressed in tumor cells and associated with tumor proliferation. Knockdown of tumor cell HMGB1 by shRNA did not inhibit tumor cell growth, whereas CD8+ T cell sensitized by tumor-specific IFN-g and TNF-a can be activated with attenuated functional Tregs (122). Since Foxp3 contributes to Treg development and activation, knockdown of Foxp3 on Tregs by shRNA successfully suppressed tumor growth (123). Transforming growth factor-beta (TGF-b) and IL-10 expressed by tumors could also trigger Tregs activation. siRNA against TGF-b decreased Treg level and enhanced DC-based cancer vaccine in mice with “cold” melanoma tumor (124), and siRNA targeting Interleukin-10 receptor alpha (IL-10Ra) initiated tumor-specific CD8+ T cell immune responses (125). When used in combination, siRNA against TGF-b/IL-10Ra significantly upregulated MHC1, CD40, CD80, CD86, and CCR7 in DCs which induced the strongest antitumor effects in immune-resistant murine models (125). Direct downregulation of chemokines such as CCL22 and CCL17 by siRNA also can decrease Treg recruited by monocyte-derived DCs (MoDCs) with lower CD4+/CD8+ ratios. Furthermore, intratumoral injection of MoDCs treated with siRNA against CCL22/CCL17 significantly reduced the Tregs population and increased CD8+ infiltration into TME (126).

miRNAs also indirectly facilitate the interaction between tumors and host immune systems which can be targeted for either downregulation or replacement by RNAi. Oncogenic miRNAs have been reported to promote tumor progression by inducing M2-like TAM in TMEs (miR-155 (127), miR-21 (128), miR-23a/27a/24-2 cluster (127)) and eliminating NK-mediated killing (miR-20a, miR-93, miR-520d, miR-106b, miR-373, miR-302c, miR-520c, miR-20b, miR-519a-3p (129, 130)), while tumor-suppressive miRNAs could inhibit tumor growth by shifting M2- to M1-TAM (miR-19a-3p (131)), inducing pro-inflammatory cytokines (miR-240-5p (132)), blocking immune checkpoint (miR-3609 (133)), and preventing the recruitment of immune suppressive cells (miR-126 (134)). Although current miRNA therapeutics against cancer remain focusing on the oncogenes which directly affect cell proliferation, embedding miRNA sequence with such antitumor immunomodulatory effects could also be an exciting strategy for RNAi based cancer immunotherapy.

## From tumor antigens to mRNA-based therapies

Most of the immunotherapies against cancer rely on the delivery of tumor-associated antigens (TAAs) and tumor-specific antigens (TSAs). Neoantigens expressed in the mutated tumor microenvironment would allow the development of personalized cancer vaccines with patient-specific neoepitopes. Tumor antigens against cancer mainly depend on the delivery of tumor antigen peptides or coding mRNAs (8, 135). Delivery of long synthetic peptides from TAAs/TSAs using liposomes can greatly protect the peptides from degradation with more access to the APCs (135). Several studies have shown that liposomes encapsulating T cell epitopes against LHR hormone (136), Pan HLA-DR epitope peptides (137), HER2/neu peptide (138), and KRAS G12 mutant peptides (139) could stimulate CD4+ T cell responses against tumors. When embedding cationic lipids or external Fc receptor ligands, liposomes with tumor antigens could show greater cell uptake by APCs[141,145]. However, the identification and manufacturing of tumor antigen peptides are time-consuming. Compared with tumor antigens in the forms of pDNA or peptides, mRNA has emerged as a new potent and flexible platform for cancer immunotherapies (7). mRNA therapies can encode protein sequences and stimulate innate immune systems. More importantly, large-scale production of mRNA is relatively simple and inexpensive in a cell-free environment, and mRNA can be easily encapsulated into lipid-based NPs through electrostatic interaction when mixing the lipids and mRNAs stock solutions, all of which make the manufacturing process of mRNA in a standardized and controlled condition (8).

### Preclinical studies of mRNA/lipid-based NP therapies

**Figure 4:**
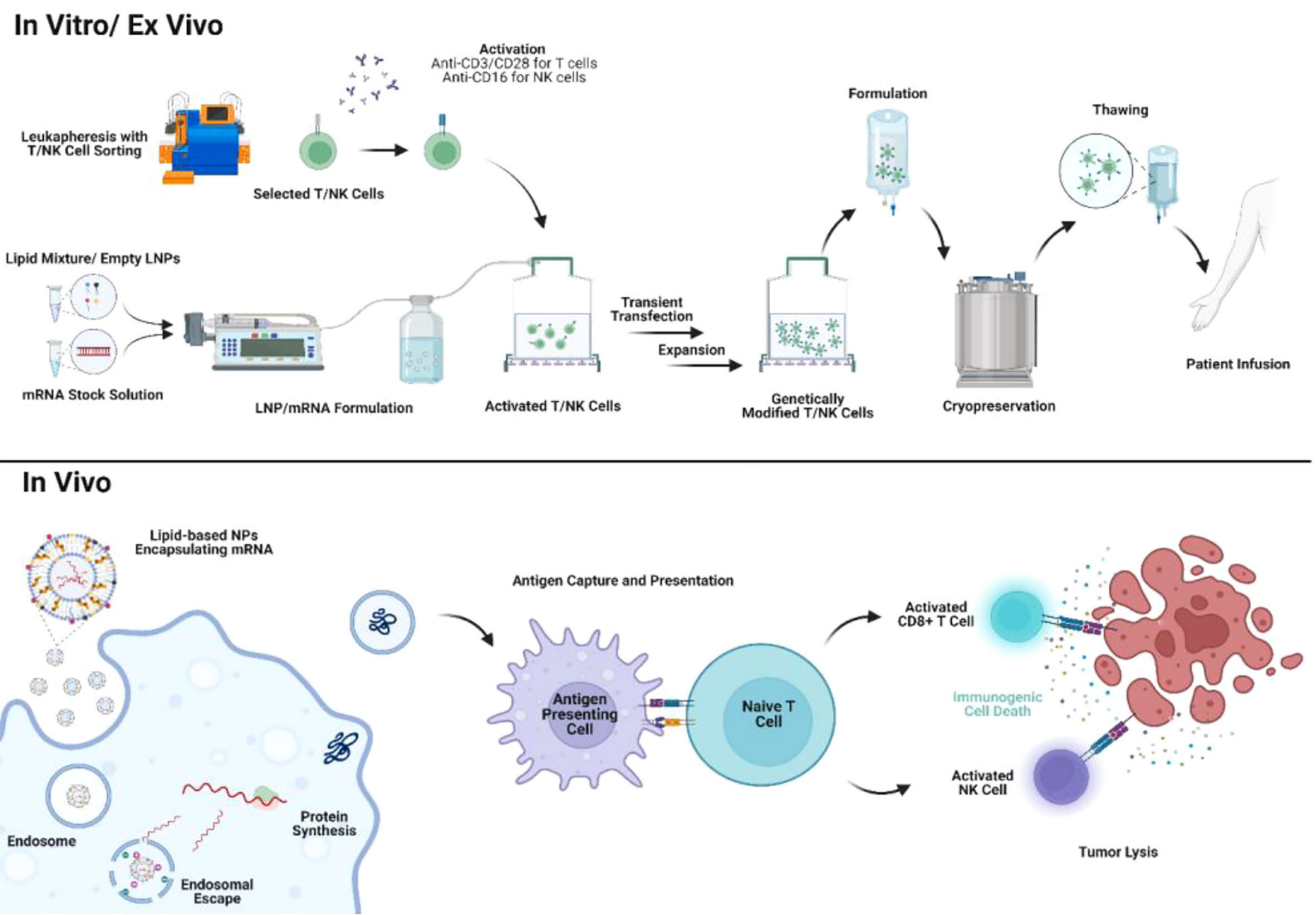
Application of mRNA/lipid-based NPs in mRNA therapies and cell therapies. mRNA/lipid-based NPs can be utilized for engineering cell therapy *ex vivo* with shortened manufacturing timeline and reduced physiological side effects. In addition, mRNA/lipid-based NPs can also be directly delivered *in vivo* to either engineer immune cells or as exogenous tumor antigens.

mRNA vaccines could stimulate an antigen-specific immune response when the encoded antigen is translated to proteins in the cytosol of APCs. The expressed proteins are processed by APCs and presented on MHC1 to CD8+ T cells, stimulating cell-mediated immune responses (**Figure 4**). Reports also showed that the fusion of mRNA-encoded antigen to MHCII trafficking signals derived from lysosomal proteins could also induce supportive CD4+ T helper cell response which is crucial in cancer immunotherapy (140, 141). In addition, the immunogenicity of mRNA structures may activate signals through TLR3, TLR7, and TLR8 which enhance innate immune responses (142). From this aspect, co-administration of mRNA/lipid-based NPs with adjuvants may enhance the stimulation of immune responses (143). For example, co-delivering the nucleoside-modified mRNA with a TLR4 agonist (monophosphoryl lipid A; MPLA) inside DOTAP–cholesterol mRNA lipoplexes induced innate immunity and allowed high antigen expression *in vivo (* 143). LNPs encapsulating mRNA encoding OVA also showed significant tumor prevention when combined with TLR1/2 agonist (92). Intramuscular immunization of OVA mRNA using Pam3-LNP in mice resulted in high expression of tumor antigens with enhanced cellular immune stimulation (92). In addition, the efficiency of mRNA–LNPs encoding interleukin-12, an example of cytokines with anticancer activity, was examined by the group Lai et al. for the suppression of tumor growth in transgenic mouse models of hepatocellular carcinoma (HCC) (144). miRNA target sites can be incorporated in modified mRNAs encoding toxic or apoptotic proteins like caspase or p53 upregulated modulator of apoptosis (PUMA) (145). The presence of miRNA binding sites will allow the targeting of miRNAs that are present only in healthy cells and then enable these cells to recognize and degrade toxic mRNA. It was found that intratumoral administration of LNPs loaded with these miRNA–mRNA combination sequences in mice prevented the expression of toxic proteins from the mRNA of healthy cells but selectively triggered apoptosis in tumor cells without causing systemic toxicity (145).

To induce a strong cytotoxic CD8 T cell response, Oberli et al. developed LNPs for the delivery of an mRNA vaccine encoding the model immunology protein, ovalbumin (OVA) (146). The authors identified an optimum formulation that contained an ionizable lipid (cKK-E12) and an additive (sodium lauryl sulfate). The optimal formulation showed increased T cell response upon reducing the molar ratio of cKK-E12 from 35% to 10%. Immunization of model mice with transgenic OVA-expressing tumor or with aggressive B16F10 melanoma using the formulated mRNA vaccine encoding the corresponding antigens resulted in strong CD8 T cell immunity activation in addition to slow tumor growth, shrinkage of tumor, and consequently, extended survival of treated mice (146). However, the increased CD8 T cell responses with decreased cKK-E12 suggest that ionizable lipids or cationic lipids themselves may have effects on directing T cell functions regardless of their main roles in binding with mRNAs.

### Clinical investigations on mRNA/lipid-based NP therapies

The safety, immunogenicity, and tolerability of the first personalized IVAC MUTANOME (BioNTech RNA Pharmaceuticals GmbH), a poly-neoepitope-coding RNA vaccine, have been evaluated in phase I clinical trials (NCT02035956) targeting mutant neoantigens for the treatment of patients with melanoma. A strong immune response against the vaccine antigens was observed. T cell response was also generated against 60% of the 125 selected neoepitopes with no adverse drug reactions, indicating good tolerability of the vaccine by enrolled patients (147). Many other personalized mRNA cancer vaccines encoding different antigens have been formulated in lipid nanosystems and have already entered clinical stages (NCT03897881, NCT02316457, NCT03313778, NCT03480152, NCT03323398) (148, 149). In a phase II study using mRNA-4157 and pembrolizumab treating melanoma patients (NCT03897881), 14 out of 16 patients in the mRNA-4157 monotherapy group remained disease-free during the study, with a median follow-up time of 8 months. In the combination group, the overall response rate in the cohort (human papillomavirus-negative, immune checkpoint inhibitor-naive, head and neck squamous cell carcinomas) was 50% and the median progression-free survival was 9.8 months (150, 151). mRNA-4157 used for the treatment of gastrointestinal cancers is also in progress (NCT03480152) (152). In another phase I/II trial (NCT03323398), the safety and efficacy of mRNA encoding human OX40L (mRNA-2416) was investigated in combination with durvalumab for the treatment of ovarian cancers and other solid tumors (153). mRNA-2416 was intratumorally administered every 2 weeks for up to 12 doses with four dose levels from 1 to 8 mg. The injected lesions showed an increase in OX40L expression and enhanced T cell activation (153). In a phase I dose-escalation study (NCT03739931), mRNA-2752 encoding human OX40L, IL-23, and IL36y was designed to induce a pro-inflammatory TME and simultaneously strengthen T cell expansion as well as memory responses. mRNA-2752 was intratumorally administered every 2 weeks for up to seven doses, alone or in combination with the infusion of durvalumab. In the 22 patients (monotherapy: n = 15; combination: n = 7), six had stable disease, one had partial responses with 52% tumor reduction and five showed tumor shrinkage in treated and/or untreated sites (154, 155).

In a phase I study (NCT02410733), FixVac, a complex of RNA/lipoplexes against malignant melanoma TAAs New York-ESO 1, tyrosinase, melanoma-associated antigen A3 (MAGE-A3), and trans-membrane phosphatase with tensin homology (TPTE) showed the metabolic activity of the spleen increased post the sixth immunization, indicating the targeted delivery of an mRNA-LNP encoding four non-mutated melanoma antigens and activation of resident immune cells (156). Within the same study, a combination of FixVac with an anti-programmed cell death protein 1 (PD1) antibody augmented the antitumor effect of FixVac, resulting in an over 35% tumor regression rate in immune checkpoint inhibitor-experienced patients (156). The results suggest that LNP-based mRNA therapies can be of general utility for non-mutant TAAs in patients who experienced ICI treatments.

## Gene engineering for cell therapies

The development of cell therapies can be traced back to vaccination with DCs including TAAs mRNA transfected by cationic lipids in the late 1990s (157). However, TAA mRNA transfection is inefficient with only cationic lipids. With optimized lipid-based NP formulation, scientists are now able to successfully engineer DCs as cancer vaccines *in vitro* and *in vivo* (158, 159). Another study indicated that *in vivo* DC engineering by directly administering lipid-based NPs with mRNA encoding antitumor immune epitopes exhibited similar antitumor efficacy compared with *in vitro* DC engineering before infusion (160). Same as APCs, *in vivo* reprogramming macrophages with lipid-based NPs containing mRNA encoding M1-polarization factors also exhibit antitumor activity in multiple murine cancer models (161). Macrophages could horizontally transfer genes into TMEs (162). In other words, it is promising to develop lipid-based NPs containing mRNA or RNAi which could co-target macrophages and tumor cells.

Recently, personalized adoptive cell therapy showed great promise against non-solid tumors in clinical trials. Adoptive cell therapy includes TIL therapy, engineered T cell receptor therapy (TCR-T), CAR-T cell therapy, and NK cell therapy. Despite their tremendous potential, adoptive cell therapies also raised concerns regarding the unwanted immunological side effects and insertional mutagenesis in the human genome due to the use of viral vectors for *ex vivo* cell engineering (163, 164). In addition, complicated manufacturing protocols and high costs can also impede the application of CAR-T in a broader patient population (163, 164). Therefore, novel *ex vivo* transfection technologies are needed for a safer and affordable adoptive cell therapy (165–167). Lipid-based NPs containing coding DNA or mRNA showed outstanding efficacy in transient transfection in preclinical studies. Lipid-based NPs encapsulating mRNA can be formulated by simple rapid mixing where a lipid stock in the ethanol phase is mixed with RNA in the aqueous phase through microfluidics or syringe pumps (168). mRNA/lipid-based NPs could be developed instantly when activated T cells or NK cells are ready for cell engineering *in vitro* or *ex vivo*. Therefore, the rationale for selecting the components of lipid-based NPs for cell engineering can mainly focus on its transfection efficiency of payloads. In addition, lipid-based NPs are generally considered with low cytotoxicity (169). Therefore, the processes of gene transfection and T/NK cell activation could be simultaneous (**Figure 4**). The straightforward formulation and low toxicity of lipid-based NPs encapsulating mRNA would greatly reduce the manufacturing cost and time for engineering cell therapies. McKinlay et al. first developed a combinatorial library in screening efficient mRNA transfection approach to lymphocytes (170). Soon, Billingsley et al. reported the development of ionizable LNP encapsulating CAR for *ex vivo* T cell engineering which first demonstrated the *ex vivo* engineered CAR-T by LNPs exhibited similar tumor-killing activity compared with lentivirus-engineered CAR-T (171). Lipid-based NPs/mRNA transfection strategy is also used in NK cell engineering. Chandrasekaran et al. first reported the development of super NK cells engineered by liposomes containing apoptosis-inducing ligand TRAIL (172). TRAIL-engineered NK cells exhibited strong tumor-killing activity by inducing apoptosis in tumor-draining lymph nodes *in vivo (* 172). Another study also reported that liposomes can co-deliver CAR mRNA and paclitaxel to NK cells (173). CAR-engineered NK cells showed significant cytotoxicity to HER2+/CD19+ tumors *in vivo* with simultaneously paclitaxel release (173). Lipid-based NPs also demonstrated efficient CAR engineering to NK cells with preserved cell viability and minimal changes in NK phenotype and function compared with the traditional electroporation method (174).

## Perspectives on clinical translation

The main efforts of lipid-based NPs in clinical trials against cancer are to deliver chemotherapies and therapeutic nucleic acids as discussed in previous sections. However, the number of lipid-based NPs formulations successfully approved in the market is still limited, suggesting that the development of lipid-based NPs remains certain barrier in the translation from preclinical animal models to humans.

Non-specific gene regulation by lipid-based NPs may take place when lipid-based NPs are used for gene delivery. It is noticeable that although the structure of lipid-based NPs is almost similar to those of lipid nanoparticles or micelles expressed by cells, the synthesized functional lipids, such as cationic or ionizable lipids, are still considered exogenous lipid-like materials which may be potentially recognized by PRRs on the cell surface. The high amount of exogenous functional lipids within lipid-based NPs may further trigger unwanted cell cycle arrest, cellular metabolism, or immune responses, which would disrupt the targeted gene regulation by nucleic acid payloads in lipid-based NPs (175, 176). When lipid-based NPs are delivered to immune cells, multiple vehicle-based immune activations may be triggered. Not only functional lipids may serve as ligands for inflammatory signals (175), but also lipid solvents, such as ethanol and chloroform, during lipid-based NP manufacturing would modulate the function of immature innate immune cells (177). In addition, PEG lipids could induce the secretion of anti-PEG immunoglobulins by B cells (178). Although the effect of anti-PEG immunoglobulins on tumor microenvironment is yet to be determined, the anti-PEG immunoglobulins would increase the clearance of lipid-based NPs in systemic fluids which would decrease the antitumor potency. Non-specific gene regulation and immune activation may potentially reduce the therapeutic efficacy whereas increasing the dose of lipid-based NPs to patients would eventually result in toxicities or irAEs. To minimize the non-specific gene regulation and immune activation by lipid-based NPs, the off-target effects of functional lipids should be characterized, and the manufacturing procedures should be optimized to purify the excipients in lipid-based NPs.

Although lipid-based NPs are believed to passively target and accumulate in tumors through the enhanced permeability and retention (EPR) effect, a growing body of research has revealed that lipid-based NPs, without any ligand-modification for active targeting, mainly accumulate in the liver upon systemic administration (179). Such effects are mainly due to discontinuous vasculature, decreased blood flow rates, and an abundance of phagocytic cell types in the liver microenvironment, which might cause liver toxicities and loss of *in-situ* therapeutic efficacy (179). Therefore, lipid-based NPs should be further engineered to specifically target tissues or cell populations to reduce liver accumulation and increase therapeutic efficacy. **Table 1**.

**Table 1:** Cases of immunological regulation of chemotherapies using lipid-based NPs as delivery system.

Drug Name	Commercial Name	Lipid-based NPs	Indications	Effects on Immune System
Approved in US and EU				
Doxorubicin	Doxil^®^	HSPC: cholesterol: DSPE-PEG (180)	Breast neoplasms, multiple myeloma, ovarian neoplasms, Kaposi’s sarcoma	MDSCs↓, DCs↑, IL-1β↑, γδ T cells↑, CD8+ T cells↑.
	Myocet^®^	EPC: Cholesterol (180)	Breast neoplasms	
	Lipodox^®^	HSPC: Cholesterol: DSPE-PEG (180)	Breast neoplasms	
Daunorubicin	DaunoXome^®^	DSPC: Cholesterol (180)	Cancer advanced HIV-associated Kaposi’s sarcoma	IL-1β↑
	Vyxeos^®^ (CPX-351) (Daunorubicin Cytarabine)	DSPC: DSPG: Cholesterol (180)	Acute myeloid leukemia	
Vincristine	Marqibo^®^	SPH: Cholesterol (180)	Philadelphia chromosome-negative acute lymphoblastic leukemia, hematologic malignancies and solid tumors	PD-L1↑, sensitive to DCs.
Irinotecan	Onivyde^®^	DSPC: Cholesterol: DSPE-PEG (180)	Metastatic pancreatic cancer	Tregs↓, MHC1↑, PD-L1↑.
Cisplatin	Lipoplatin™ Nanoplatin™	DPPG: soy PC: MPEG-DSPE: Cholesterol (180)	Pancreatic cancer Lung cancer	CCL5↑, CXCL9↑, and CXCL10↑
Newly in Clinical Trial				
Mitoxantrone	Liposome Encapsulated Mitoxantrone (LEM)	DOPC: Cholesterol: Tetramyristol cardiolipin (181)	Tumors	Calreticulin (CRT)↑
CKD-602	S-CKD602	MPEG Lipid Conjugation (182)	Advanced malignancies	NA
Topotecan	INX-0076	Cholesterol: Sphingomyelin (180)	Advanced solid tumours	MHC1↑, Fas↑, sensitive to effector T cells.
	LEP-ETU	DOPC: Cholesterol: Cardiolipin (183)	Advanced cancer (Neoplasm), metastatic breast cancer	
Paclitaxel	MBP-426^®^	Transferrin: NG-DOPE (184)	Solid Tumors	TILs↑, MHC1↑, CD8+/CD4+ T cells↑, Tregs↓, TLR4↑
Oxaliplatin	OSI-211	HSPC: Cholesterol (185)	Recurrent small cell lung cancer (SCLC)	MHC1↑, Tregs↓, MDSCs↓, TAMs↓

↑represents positive regulation; ↓represents negative regulation.

## Conclusion

Lipid-based NPs represent the most advanced and widely applicable delivery vehicles for small molecules and nucleic acids. In cancer immunotherapy, lipid-based NPs not only could deliver small molecules and mRNA therapies *in vivo* to achieve the enormous antitumor activity but also are capable of *ex vivo* engineering cancer cell therapies with comparable efficiency compared to other non-viral or viral vectors. However, the formulation and large-scale manufacturing process of lipid-based NPs need to be optimized from industrial aspects. Depending on the specific drug payloads and applications, different helper lipids and cationic/ionizable lipids need to be selected to address specific challenges to immunotherapeutic delivery. For small-molecule chemotherapies or ligands for surface PPRs, lipid-based NPs with high colloidal stability should be designed to prolong their systemic retention time. Therefore, lipids with higher T_m_ are preferred due to their long-term stability in storage and the systemic fluid. For RNAi, mRNA, and ligands targeting intracellular PPRs, lipid-based NPs should be designed to satisfy membrane fusion and endosomal escape, such as embedding PE-based lipids and decreasing PEG-lipids, where the stability for lipid-based NPs may be compromised. Fortunately, the challenges for colloidal stability and *in vivo* side effects by lipid-based NPs may be bypassed in the application of cell therapies where lipid-based NPs containing mRNAs can be formulated and transfected into T/NK cells *ex vivo* in a real-time manner. However, due to the transient gene transfection by lipid-based NPs/mRNA, the durability of tumor-killing activities by engineered T/NK cells need to be further evaluated thoroughly. In addition, there are stilling remaining concerns for the delivery of lipid-based NPs, such as immunogenicity and tissue accumulation. Therefore, efforts should also be made on developing lipid-based NPs targeting specific organs or cell-populations as well as utilizing biodegradable formulation components with low immunogenicity (186, 187).

Moreover, the therapeutic payloads need to be designed for optimal lipid-based NP delivery efficiency and antitumor immunity. Small molecule drugs can be designed with ionizable structures to be stably encapsulated into the lipid-based NPs, and their mechanism of actions should be well identified without off-target effects such as inducing immunosuppression or autoreactive immune disorders (188). Nucleic acid drugs can be chemically modified to enhance their stabilities, and their sequence should be optimized to avoid off-target gene regulations (189). For mRNA drugs specifically, the translation efficiency can be increased by engineering their coding sequences, 5’ cap, 3’ poly(A) tail, and untranslated regions (UTRs) (18). Beside the single drug delivery, multiple immunostimulatory factors or epitopes could be delivered together within one NPs to achieve the optimal antitumor activity. For example, PPR agonists could be co-delivered with mRNA encoding TAAs to maximize tumor-specific antigen presentation; mRNA encoding Th1 epitopes in combination with RNAi silencing immune-suppressive genes may enhance CD8+ T cell and NK cell activation; Co-delivering of mRNA encoding CAR and chemotherapies in T/NK cells may also increase tumor killing activities. It is also noticeable that cancer immunotherapies only showed potency in patients with “hot” tumors where immune cells can rapidly infiltrate into TME. Systemic immunostimulatory agents, such as PPR agonists, may be involved in developing immunotherapies for a broader patient population. With the optimized formulation and proper payload recipes, lipid-based NP medicines would not only expand cancer immunotherapies to a broader range of patient populations but also improve health care in other diseases.

## Author contributions

Conceptualization, ZZ; writing—original draft preparation, ZZ and SY; writing—review and editing, ZZ, SY, YH, XZ, and RL. All authors have read and agreed to the published version of the manuscript.

## Acknowledgments

All figures are created with https://biorender.com/.

## Conflict of interest

YH and XZ are employed by The Whiteoak Group, Inc.

The remaining author declares that the research was conducted in the absence of any commercial or financial relationships that could be construed as a potential conflict of interest.

## Publisher’s note

All claims expressed in this article are solely those of the authors and do not necessarily represent those of their affiliated organizations, or those of the publisher, the editors and the reviewers. Any product that may be evaluated in this article, or claim that may be made by its manufacturer, is not guaranteed or endorsed by the publisher.
