# Supplementary material for: Application of lipid-based nanoparticles in cancer immunotherapy
Source: Front Immunol. 2022 Aug 8;13:967505. doi: 10.3389/fimmu.2022.967505 (PMC9393708; doi:10.3389/fimmu.2022.967505)
Supplement: Supplementary file 1 [file DataSheet_1.docx]

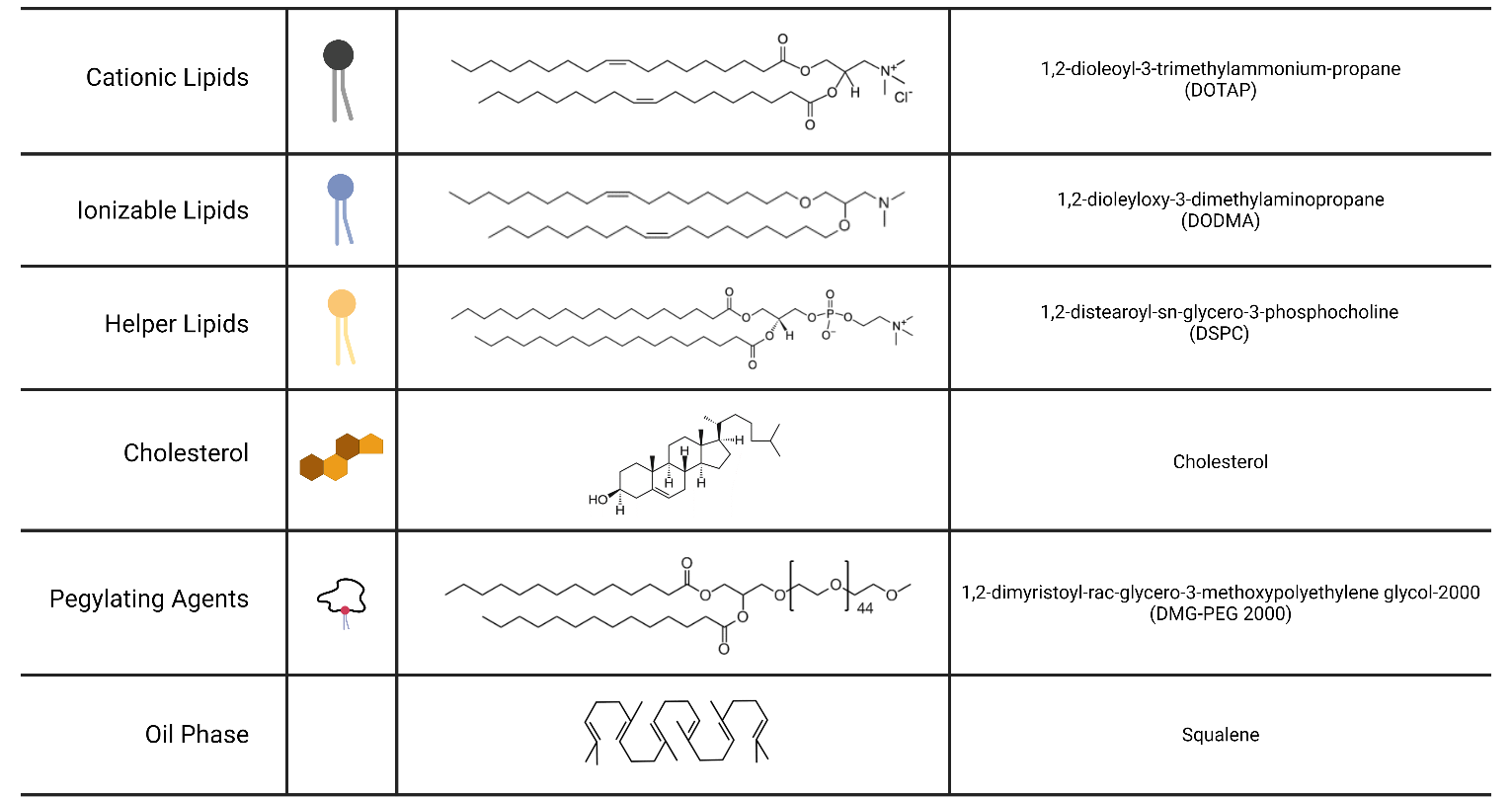


**Figure S1**. Representative structures of each lipid-based NP components.

**Table S1**. List of current ionizable lipids.

| **Abbreviation** | **Reference** | **Full Chemical Name** |
| --- | --- | --- |
| A-066 | 42 | 1-(2,3-bis(((9Z,12Z)-octadeca-9,12-dien-1-yl) oxy) propyl) pyrrolidine |
| A18 | 50 | ethyl 1-(3-(2-ethylpiperidin-1-yl) propyl)-5,5-di((Z)-heptadec-8-en-1-yl)-2,5-70 dihydro-1H-imidazole-2-carboxylate |
| A9 (Acuitas) | 48 | NA |
| ALC-0315 | 41 | ((4-hydroxybutyl) azanediyl) bis(hexane-6,1-diyl) bis(2-hexyldecanoate) |
| ATX (Arcturus) | 51 | NA |
| C12-200 | 43 | 1,1′-((2-(4-(2-((2-(bis(2-hydroxydodecyl) amino) ethyl) (2-hydroxydodecyl) amino) ethyl) piperazin-1-yl) ethyl) azanediyl) bis(dodecan-2-ol) |
| cKK-E12 | 44 | 3,6-bis(4-(bis(2-hydroxydodecyl) amino) butyl) piperazine-2,5-dione |
| DLinDMA | 38 | 1,2-dilinoleyloxy-N, N-dimethyl-3-aminopropane |
| DLin-KC2-DMA, KC2 | 36 | N, N-dimethyl-2,2-di-(9Z,12Z)-9,12-octadecadien-1-yl-1,3-dioxolane-4-ethanamine |
| DLin-MC3-DMA, MC3 | 35 | 4-(dimethylamino)-butanoic acid |
| DODAP | 37 | 1,2-dioleoyl-3-dimethylammonium-propane |
| DODMA | 37 | 1,2-dioleyloxy-3-dimethylaminopropane |
| FTT5 | 46 | Hexa(octan-3-yl) 9,9′,9″,9‴,9″″,9‴″-((((benzene-1,3,5-tricarbonyl) ris(azanediyl)) tris (propane-3,1-diyl)) tris(azanetriyl)) hexanonanoate |
| L319 | 39 | di((Z)-non-2-en-1-yl) 9-((4-(dimethylamino) butanoyl) oxy) heptadecanedioate |
| Lipid 5 | 49 | heptadecan-9-yl 8-((2-hydroxyethyl) (8-(nonyloxy)-8-oxooctyl) amino) octanoate |
| LP01 (Intellia Therapeutics) | 52 | NA |
| SM-102 | 40 | heptadecan-9-yl 8-((2-hydroxyethyl) (6-oxo-6-(undecyloxy) hexyl) amino) octanoate |
| ssPalm | 47 | α-D-Tocopherolsuccinoyl |
| TT3 | 45 | N1, N3, N5-tris(3-(didodecylamino) propyl) benzene-1,3,5-tricarboxamide |
